# Increased Extent of and Risk Factors for Pandemic (H1N1) 2009 and Seasonal Influenza among Children, Israel

**DOI:** 10.3201/eid1709.102022

**Published:** 2011-09

**Authors:** Dan Engelhard, Michal Bromberg, Diana Averbuch, Ariel Tenenbaum, Daniele Goldmann, Marina Kunin, Einat Shmueli, Ido Yatsiv, Michael Weintraub, Michal Mandelboim, Nurith Strauss-Liviatan, Emilia Anis, Ella Mendelson, Tamy Shohat, Dana G. Wolf, Mervyn Shapiro, Itamar Grotto

**Affiliations:** Author affiliations: Hadassah-Hebrew University Hospital, Ein Kerem, Jerusalem, Israel (D. Engelhard, D. Averbuch, E. Shmueli, I. Yatsiv, M. Weintraub, N. Strauss-Liviatan, D.G. Wolf);; Monash University, Melbourne, Victoria, Australia (D. Engelhard, M. Kunin);; Israel Center for Disease Control, Tel Hashomer, Israel (M. Bromberg, T. Shohat);; Hadassah-Hebrew University Hospital, Mount-Scopus, Jerusalem (A. Tenebaum);; Public Health Services, Health Ministry, Jerusalem (D. Goldmann, E. Anis, I. Grotto);; Sheba Medical Center, Tel Hashomer (M. Mandelboim, E. Mendelson);; Tel Aviv University, Tel Aviv, Israel (T. Shohat); Tel Aviv Sourasky Medical Center, Tel Aviv (M. Shapiro);; Ben-Gurion University of the Negev, Beersheba, Israel (I. Grotto)

**Keywords:** viruses, pandemic (H1N1) 2009, children, epidemiology, influenza-like illness, ILI, sentinels, clinical characteristics, mortality, influenza, Israel, dispatch

## Abstract

During the pandemic (H1N1) 2009 outbreak in Israel, incidence rates among children were 2× higher than that of the previous 4 influenza seasons; hospitalization rates were 5× higher. Children hospitalized for pandemic (H1N1) 2009 were older and had more underlying chronic diseases than those hospitalized for seasonal influenza.

We compared the extent and pattern of pandemic (H1N1) 2009 with the previous 4 influenza seasons (2005–2009) among Israel’s child population, both for community-based surveillance and pediatric hospitalizations. We also sought a possible association between the pandemic waves and schools closure. The study was approved by the Institutional Review Board Committee of Hadassah Medical Center.

## The Study

Israel’s Center for Disease Control seasonal influenza surveillance system operated throughout our 5-year study. The system is based primarily on 1) anonymous patient visits for influenza-like illnesses (ILI) to Maccabi Community Clinics, Israel’s second largest health maintenance organization, insuring ≈1 of every 4 Israelis; and 2) nasopharyngeal swabs from sample ILI patients at designated sentinel clinics countrywide. ILI was defined as fever (>37.8°C) with >1 of the following: cough, coryza, sore throat, or myalgia. Swab samples were tested for influenza viruses at the Health Ministry’s Central Virology Laboratory (*1*) by using multiplex real-time reverse transcription PCR (RT-PCR) (TaqMan chemistry quantitative RT-PCR) (*2*).

**Figure 1:**
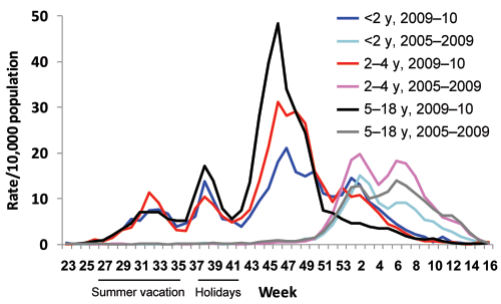
Rates of weekly visits to community health clinics for influenza-like illness, by age group, June 2009–April 2010, compared with the 2005–2009 average (Maccabi Health Services), with school holidays indicated, Israel.

ILI rates constituted 3 escalating waves of infection, all at times atypical for seasonal influenza (Figure 1). The first peaked early August (week 32). Israel’s schools close July/August, but children stay together in summer frameworks during July. Wave 2 peaked mid-September (week 38), 2 weeks into the school year, declining when schools closed for holidays until the end of week 41. During week 42, the third, largest wave began, peaking mid-November (week 46).

The cumulative incidence (cases/10,000 population) of ILI in children 0–18 years of age during the pandemic (week 25, 2009 to week 7, 2010) was 369.3 (95% confidence interval [CI] 365.7–373.1), far higher than average rates documented in earlier influenza seasons (143.4, 95% CI 140.7–146.2). Incidence was 295.8 (95% CI 285.7–306.1) for children <2 years of age, 347.1 (95% CI 338.4–355.9) for children 2–4 years of age, and 389.4 (95% CI 382.5–391.4) for children 5–18 years of age, compared with 107.8 (95% CI 100.9–115.1), 179.5 (95% CI 172.5–186.7), and 140.2 (95% CI 140.7–146.2), respectively, for each age group in earlier seasons.

**Figure 2:**
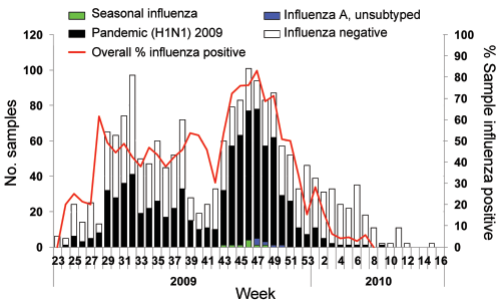
Positive influenza samples among total samples collected by the sentinel network, children 0–18 years of age, Israel, June 2009–April 2010.

Israel identified its first pediatric pandemic (H1N1) 2009 cases in June 2009 (week 24) and recorded local transmission the following week (Figure 2). During weeks 28–43, the weekly percentage of positive influenza samples among children was 40%–60%, peaking at 70%–80% during weeks 44–49 (late October to early December). This finding correlates with the return to school for a continuous period.

Pandemic (H1N1) 2009 in Israel was present for 36 weeks; the overall percentage of positive influenza samples among children was 50%. Earlier influenza seasons were shorter (13–22 weeks) but with similar overall positive percentages (52%–57%) of influenza A/B. Most (98%) of the influenza cases during 2009 were the pandemic strain; in previous years a mixture of various influenza A/H1N1, H3N2, and B strains occurred.

We compared hospitalization of children with laboratory-confirmed influenza infection during the pandemic with the previous 4 influenza seasons in the pediatric departments of Hadassah’s 2 hospitals in Jerusalem. These departments provide primary medical care for ≈250,000 children (1 of every 10 children in Israel), as well as tertiary care for chronic diseases. We performed our study at these hospitals because respiratory specimens were routinely taken year-round for laboratory confirmation from all children with suspected influenza or respiratory virus infection during the 5-year study. Direct immunofluorescence assay was used at Hadassah in previous years for detection of influenza and other respiratory viruses and multiplex real-time PCR (TaqMan chemistry quantitative RT-PCR) for detection of influenza viruses during the pandemic.

Findings from pandemic (H1N1) 2009 were retrospectively compared with those from previous influenza seasons. Two-sample *t*-tests and the Mann-Whitney nonparametric tests compared continuous variables. Categorical variables were compared with χ^2^ and the Fisher exact tests. All tests applied were 2-tailed; a p value <0.05 was considered statistically significant.

During June 29, 2009–January 25, 2010, a total of 127 children were admitted to Hadassah hospitals with documented pandemic (H1N1) 2009 infection; most hospitalizations (77 of the total 890 children hospitalized during that period) occurred during the third peak, October–November 2009. Table 1 summarizes the major clinical manifestations for the 127 children; 33/124 (26.6%) had leukocyte counts <5,000 cells/mm^3^. All patients received oseltamivir treatment; 1 child in whom resistance to oseltamivir developed was given zanamivir. Children >2 years of age had a significantly higher rate of underlying illness compared with children <2 years of age (49/79 [62.0%] vs. 17/48 [35.4%]; p = 0.006). All survived, including 2 children who were mechanically ventilated (11 and 25 days, respectively) and another 6 who required intensive care. Nationwide, 9 children (median age 12.5 years) died from pandemic (H1N1) 2009, a mortality rate of 3.69/1,000,000. No deaths were reported during previous influenza seasons, including among infants, for which reporting is mandatory.

**Table 1:** Major clinical manifestations in 127 children hospitalized with pandemic (H1N1) 2009 infection, Hadassah University Hospitals, Israel, June 29, 2009–January 25, 2010

Symptom/finding	No. (%) patients
Fever (>38°C)	119 (93.7)
Cough	86 (67.7)
Decreased appetite	49 (38.6)
Weakness	47 (37.0)
Rhinorrhea	43 (33.9)
Vomiting/nausea	43 (33.9)
Oxygen saturation <90%	36 (28.3)
Dyspnea	34 (26.8)
Diarrhea	28 (22.0)
Abdominal pain	27 (21.3)

In previous shorter influenza A/B seasons, fewer children were hospitalized; none were treated with antiviral agents, and statistically significant differences included age, underlying chronic diseases, underlying chronic lung disease, and neonatal fever as the initial symptom (Table 2). No significant differences were found regarding history of prematurity (<33 weeks), weight percentile, pediatric intensive care unit admission, evidence of pneumonia, oxygen saturation <90%, and leukopenia. In previous seasons, 6 nosocomial influenza infections and 2 co-infections with respiratory syncytial virus were reported; none were seen for pandemic (H1N1) 2009.

**Table 2:** Comparison of children hospitalized at Hadassah University Hospitals, Israel, during pandemic (H1N1) 2009 and during influenza seasons for 2005–2009*

Parameter	Pandemic (H1N1) 2009	Seasonal influenza	p value
No. patients during 1 season	127	Median 20 (range 18–39)†	NA
Children with influenza A/B	127/0	68/28	NA
Duration of hospitalization, wks	34	Median 16 (range 13–22)	NA
No. (%) children who received antiviral treatment	98/127 (77.2)	0/96	<0.001
Patient age, mo, median (range)	51 (0.23–197.00)	6 (0.3–206.0)	<0.001
No. (%) children with underlying chronic diseases	66 (52.0)	24 (25.0)	<0.001
No. (%) children with underlying chronic lung disease	39/127 (30.7)	14 (14.6)	0.007
No. (%) children with neonatal fever as initial symptom	6/127(4.7)	26 (27.1)	<0.001

*NA, not applicable. †Total for all 4 years = 96.

## Conclusions

Children, mainly those 5–10 years of age, were affected by pandemic (H1N1) 2009 markedly more so than by seasonal influenza, similar to results reported from the United States, Spain, and Switzerland (*3**–**6*). During the 1918 Spanish influenza pandemic, the highest incidence rates were among older children (*7*). In our study, hospitalized children infected with pandemic (H1N1) 2009 were older and findings were compatible with reports from several other countries (*8**,**9*), but findings were unlike those from Argentina, where 60% were infants (*9*). The age of children who died in Israel also underlines the impact on older children, as reported elsewhere (*10**,**11*). Although pandemic (H1N1) 2009 virus may cause severe, life-threatening disease in previously healthy children of all ages (*12*), the children we studied had significantly more underlying chronic diseases than did with children hospitalized for seasonal influenza (*13*).

We, like others (*3*), found no increase in pneumonia or pediatric intensive care unit admissions caused by pandemic (H1N1) 2009. However, this finding could be because antiviral therapy was administered during the pandemic but not in previous years; 98/127 (77.2%) of children hospitalized for pandemic (H1N1) 2009 received oseltamivir (Table 2).

The nationwide pandemic (H1N1) 2009 influenza mortality rate in Israel is similar to that reported for the United Kingdom (*14*) but cannot be compared with previous years because laboratory data are lacking and there was no requirement to report the death of children >12 months of age. Our study is limited in that it was retrospective. During the pandemic, parents were advised not to attend the clinic for mild disease, although anxiety may have increased visits. There may have been differences between diagnoses of ILI among different Maccabi physicians. The 2 hospitals studied, which represented 10% of hospitalized children, were selected not as nationally representative but because of the feasibility of viral diagnosis since 2005. Influenza detection during the pandemic in patients hospitalized at Hadassah was based on PCR; immunofluorescent antibody assay was used for previous seasons.

Awareness that pandemic influenza may have unique clinical characteristics, risk factors, and increased incidence, mainly among children 5–18 years of age, is advocated. Because school opening in late summer 2009 triggered the wave of pandemic (H1N1) 2009 influenza (*15*), closing or delaying opening schools until vaccine is available should be considered among mitigation strategies in future influenza pandemics, especially for more virulent viruses**.**
